# Molecular basis for isoform-selective inhibition of presenilin-1 by MRK-560

**DOI:** 10.1038/s41467-022-33817-5

**Published:** 2022-10-22

**Authors:** Xuefei Guo, Yumeng Wang, Jiayao Zhou, Chen Jin, Jiaoni Wang, Bojun Jia, Dan Jing, Chuangye Yan, Jianlin Lei, Rui Zhou, Yigong Shi

**Affiliations:** 1 Beijing Frontier Research Center for Biological Structure, Tsinghua-Peking Joint Center for Life Sciences, Key Laboratory for Protein Sciences of Ministry of Education, School of Life Sciences, Tsinghua University, Beijing, China; 2 Westlake Laboratory of Life Science and Biomedicine, Xihu District, Hangzhou, Zhejiang Province China; 3 Key Laboratory of Structural Biology of Zhejiang Province, School of Life Sciences, Westlake University, Xihu District, Hangzhou, Zhejiang Province China; 4 Institute of Biology, Westlake Institute for Advanced Study, Xihu District, Hangzhou, Zhejiang Province China

**Keywords:** Cryoelectron microscopy, Proteases, Membrane proteins

## Abstract

Inhibition of γ-secretase activity represents a potential therapeutic strategy for Alzheimer’s disease (AD). MRK-560 is a selective inhibitor with higher potency for Presenilin 1 (PS1) than for PS2, the two isoforms of the catalytic subunit of γ-secretase, although the underlying mechanism remains elusive. Here we report the cryo-electron microscopy (cryo-EM) structures of PS1 and PS2-containing γ-secretase complexes with and without MRK-560 at overall resolutions of 2.9-3.4 Å. MRK-560 occupies the substrate binding site of PS1, but is invisible in PS2. Structural comparison identifies Thr281 and Leu282 in PS1 to be the determinant for isoform-dependent sensitivity to MRK-560, which is confirmed by swapping experiment between PS1 and PS2. By revealing the mechanism for isoform-selective inhibition of presenilin, our work may facilitate future drug discovery targeting γ-secretase.

## Abstract (web summary)

Presenilin isoforms (PS1 and PS2) containing γ-secretase show contrasting sensitivity to MRK-560. Here, the authors determined cryo-EM structures of PS1/PS2-complexes treated with MRK-560 and identified key residues responsible for the isoform-dependent selectivity.

## Introduction

The γ-secretase complex cleaves a variety of type I transmembrane (TM) proteins, exemplified by amyloid precursor protein (APP) and Notch^1–4^. APP is initially cleaved by β-secretase to generate a carboxyl-terminal, 99 residue-containing fragment, known as APP-C99, which then undergoes sequential cleavages by γ-secretase, releasing the APP intracellular domain (AICD) and β-amyloid peptides (Aβ) in various lengths^5^. Relatively longer Aβs tend to form oligomers and amyloid plaques in brain^6–9^.

Accumulation of amyloid plaques is a hallmark of AD. Inhibition of γ-secretase activity has been explored for AD treatment. However, this strategy has thus far failed to show benefits; even worse, severe side effects were observed in clinical trials^10,11^. The side effects may be in part attributed to the nonspecific inhibition of γ-secretase’s cleavage of other substrates, especially Notch, which plays a crucial role in development and cell-fate determination^12,13^. Selective inhibition of γ-secretase cleavage of APP over Notch has been proposed to mitigate the side effects. However, APP and Notch bind γ-secretase in a similar manner, complicating the design of substrate-selective γ-secretase inhibitors (GSIs)^14,15^.

A strategy to differentially inhibit γ-secretase’ cleavage of different substrates is to target the distinct isoforms of its constituents. A γ-secretase complex comprises APH-1, Pen-2, nicastrin and the catalytic subunit presenilin^16,17^. There are mainly two isoforms of presenilin, PS1/PS2, and three isoforms of APH-1, APH-1aL/aS/b, in human. γ-Secretases that contain different isoforms of each subunit exhibit varied proteolytic activities and subcellular localization in vivo^2,18^. The PS1-complex (short for PS1-containing γ-secretase) is more catalytically active for Aβ production, as seen in a cell-based assay as well as PS1/PS2 knockout mice^19^. The PS1- and PS2 complexes contribute to ~80% and 20% of Aβ production, respectively^2^. Interestingly, they release nearly equal amount of AICD and Notch intracellular domain (NICD)^20^.

PS1 hosts more than 80% of AD pathogenic mutations, and PS2 and APP carry the rest of the identified mutations (https://www.alzforum.org/mutations/). PS1-selective inhibitors, which target aberrant cleavages of APP by PS1 mutants, may thus serve as a candidate for the treatment of AD patients bearing PS1 pathogenic mutations. In this way, Notch signaling may be preserved through PS2 cleavage^21^.

A small molecule compound, *N*-[*cis*-4-[(4-chlorophenyl) sulfonyl]-4-(2,5-difluorophenyl) cyclohexyl]-1,1,1-trifluoromethanesulfonamide (known as MRK-560), is a PS1-selective GSI that shows 37 folds higher suppression of the PS1-complex over PS2^22,23^. MRK-560 leads to reduced Aβ production in the brain. Importantly, it does not induce Notch-related side effect in wild-type (WT) mouse model^22,24^. In this work, we determined the cryo-EM structures of PS1/PS2 complexes treated with MRK-560 to elucidate the molecular basis for the isoform-specificity of MRK-560.

## Results

### MRK-560 is an isoform-selective inhibitor for PS1 and PS2

**Fig. 1:**
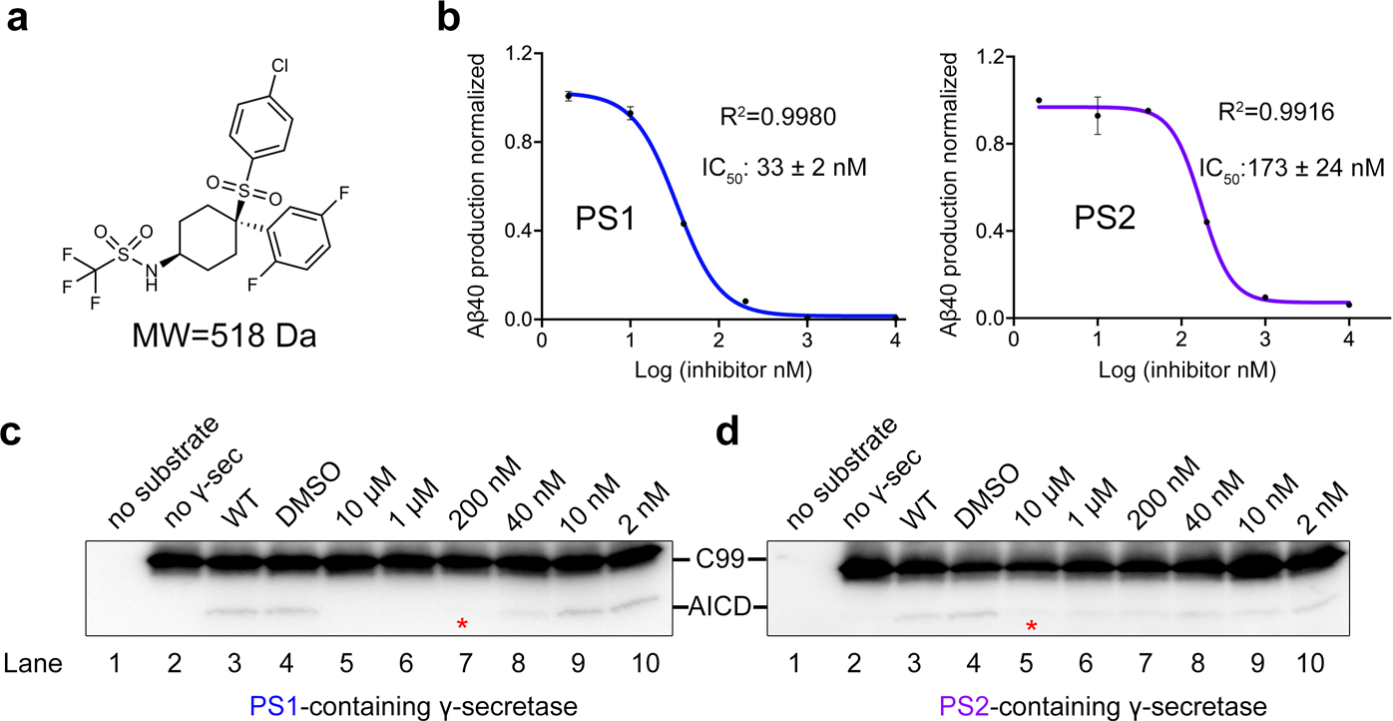
MRK-560 is a PS1-selective γ-secretase inhibitor. **a** Chemical structure of MRK-560. **b** MRK-560 preferentially inhibits the PS1-containing γ-secretase (short as the PS1-complex) in vitro. Quantification of Aβ40 production using the AlphaLISA assay yielded the IC_50_ of 33 ± 2 nM and 173 ± 24 nM for PS1- and PS2-complex, respectively. Data are presented as mean values ± SD. **c**, **d** MRK-560 is more potent for inhibiting production of the intracellular domain of APP-C99 (AICD) by PS1 than by PS2. Release of AICD in the presence of decreasing concentrations of MRK-560 from 10 μM to 2 nM was monitored by western blot with the primary monoclonal antibody against the C-terminal Myc tag. The production of AICD was quantified by ImageJ. Red asterisks indicate the first concentration where the normalized production of AICD was reduced to less than 20%. Each cleavage assay in (**b**–**d**) was repeated for three times. Error bars: SD. Source Data is available as a Source Data file.

The PS1- or PS2 complexes were expressed and purified as described^25^. To validate the inhibitory effect of MRK-560 (Fig. 1a), we reconstituted an in vitro cleavage assay using the purified PS1- or PS2 complexes. Production of Aβ40, which is the major cleavage product among the varying lengths of Aβs, and AICD, whose cleavage reflects the endopeptidase activity of γ-secretase, are measured with AlphaLISA and western blots, respectively^5^.

Consistent with previous study, MRK-560 shows more potent inhibition of the PS1-complex (Fig. 1). The IC_50_ of MRK-560 for PS1- or PS2-complex is estimated to be 33 ± 2 nM or 173 ± 24 nM, respectively (Fig. 1b). Generation of AICD by the PS1-complex is completely inhibited in the presence of 200 nM MRK-560 (Fig. 1c). In contrast, AICD produced by the PS2-complex can still be detected even in the presence of 10 μM MRK-560 (Fig. 1d).

### Selective association of MRK-560 with the PS1-complex

Atomic structures of the PS1/PS2 complexes in the presence and absence MRK-560 are needed to uncover the molecular basis for the selectivity of MRK-560. We reported the atomic structure of the PS1-complex a few years ago^25,26^. In this study, we resolved the structure of apo PS2-complex at 3.4 Å (Supplementary Figs. 1,  2,  3), which is nearly identical to that of the apo PS1-complex, with a root-mean-square deviation (RMSD) of 1.295 Å over 1164 Cα atoms (Supplementary Fig. 4a). Subtle variations occur to the catalytic residues-containing TM6 and TM7, and the loop preceding the Pro433-Ala434-Leu435 (PAL) motif, which is critical for substrate recruitment^27^ (Supplementary Fig. 4b). In the EM map of the apo PS2-complex, TM2 of PS2 is visible only in low-contour maps, indicating its intrinsic mobility (Supplementary Fig. 3a).

**Fig. 2:**
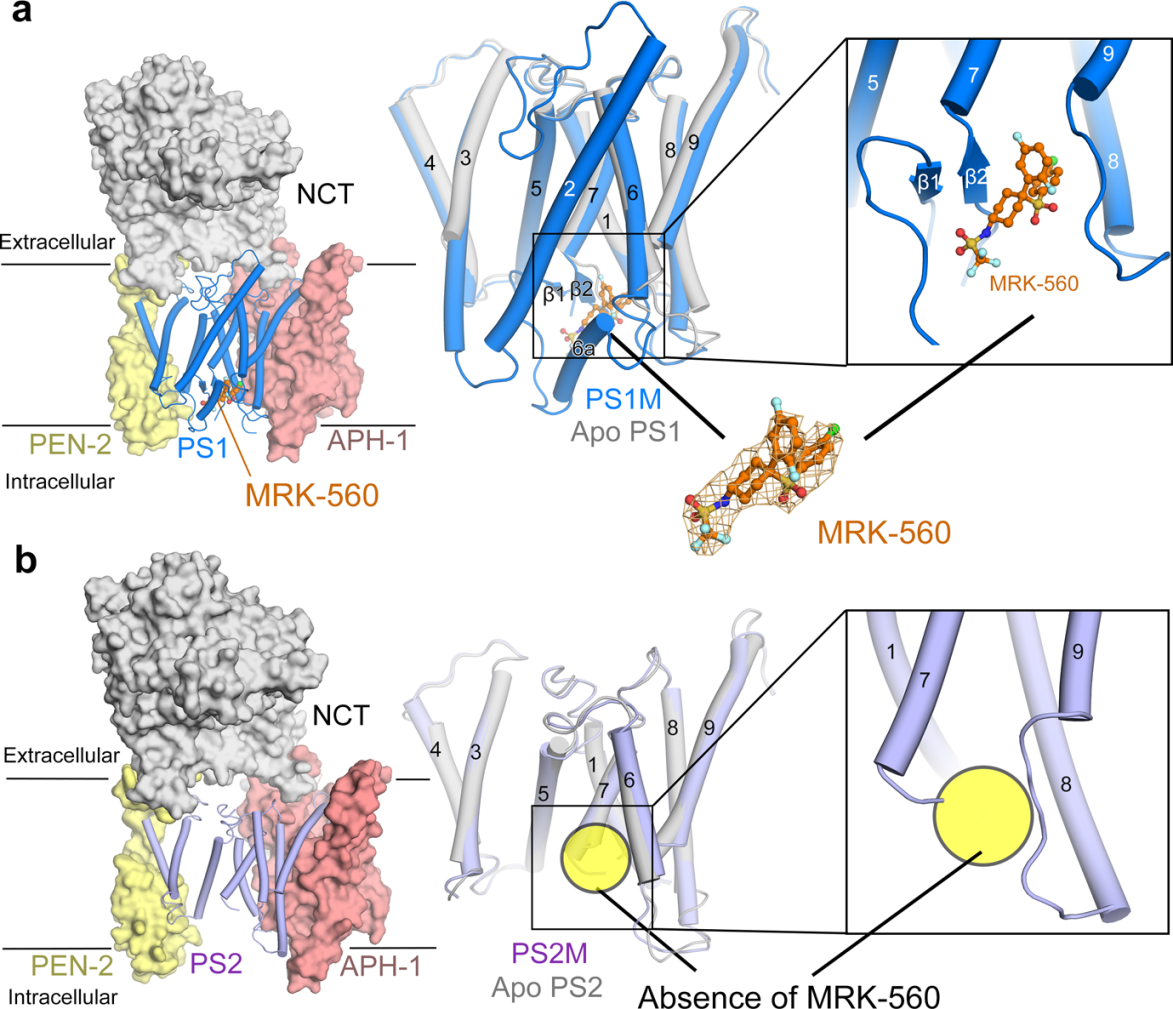
MRK-560 is observed in the PS1-complex, but not PS2. **a** Overall cryo-EM structure of the human PS1-complex bound to MRK-560. MRK-560 is shown as orange ball ad sticks. PS1 in the presence of MRK-560 is colored marine (left panel), which is aligned with PS1 in free state (middle panel). The chlorophenyl (CP) group and the difluorobenzyl (DFB) group of MRK-560 insert deeply into the binding cavity. The trifluoromethanesulfonamide (TFMS) group points towards the intracellular side. MRK-560 binding induces formation of two β-strands in PS1 (right panel). **b** Cryo-EM structures of the human PS2-complex. *Left*: The structure of PS2 treated with MRK-560 is colored light purple. *Middle*: The structure of PS2 treated with MRK-560 (PS2M) is nearly identical to that of apo-PS2 (silver). *Right*: No density corresponding to MRK-560 is observed in the PS2M map (right panel).

The PS1- and PS2 complexes were then each incubated with MRK-560, which was supplemented at a final concentration of 2 mM, before cryo-EM analysis. The 3D EM reconstructions of the PS1- and PS2 complexes treated with MRK-560, short as PS1M and PS2M, were determined at the averaged resolutions of 2.9 Å and 3.0 Å, respectively (Fig. 2 and Supplementary Figs. 1, 2,  3). In addition to the slightly higher nominal resolutions, the transmembrane region and the extracellular domain of PS1M is better resolved than those in PS2M (Supplementary Fig. 3a). In the map of PS1M, a trifurcate shaped density is discernible near the two β-strands, which are usually induced upon binding of substrates or GSIs^15,28^. MRK-560 fits the density well (Fig. 2a, Supplementary Fig. 3a, b). By contrast, no clear density is observed at the corresponding site in PS2M (Fig. 2b and Supplementary Fig. 3).

Upon binding of MRK-560, PS1 undergoes substantial conformational changes compared to the ligand-free state (Supplementary Fig. 5). Similar to other reported GSIs^28^, MRK-560 binding induces the formation of two β-strands and the movement of PAL loop. The cytosolic sequence following TM6 folds to TM6a, which is connected to TM6 through a flexible linker and bends toward the inhibitor binding site (Fig. 2a and Supplementary Fig. 5). PS2M adopts a nearly identical conformation with the apo complex (Fig. 2b, middle panel), lacking the characteristic anti-parallel β-sheet and the extension of TM6a (Fig. 2b). The parallel structural analysis demonstrates a preferential association of MRK-560 with PS1 over PS2.

### Recognition of MRK-560 by the PS1-complex

**Fig. 3:**
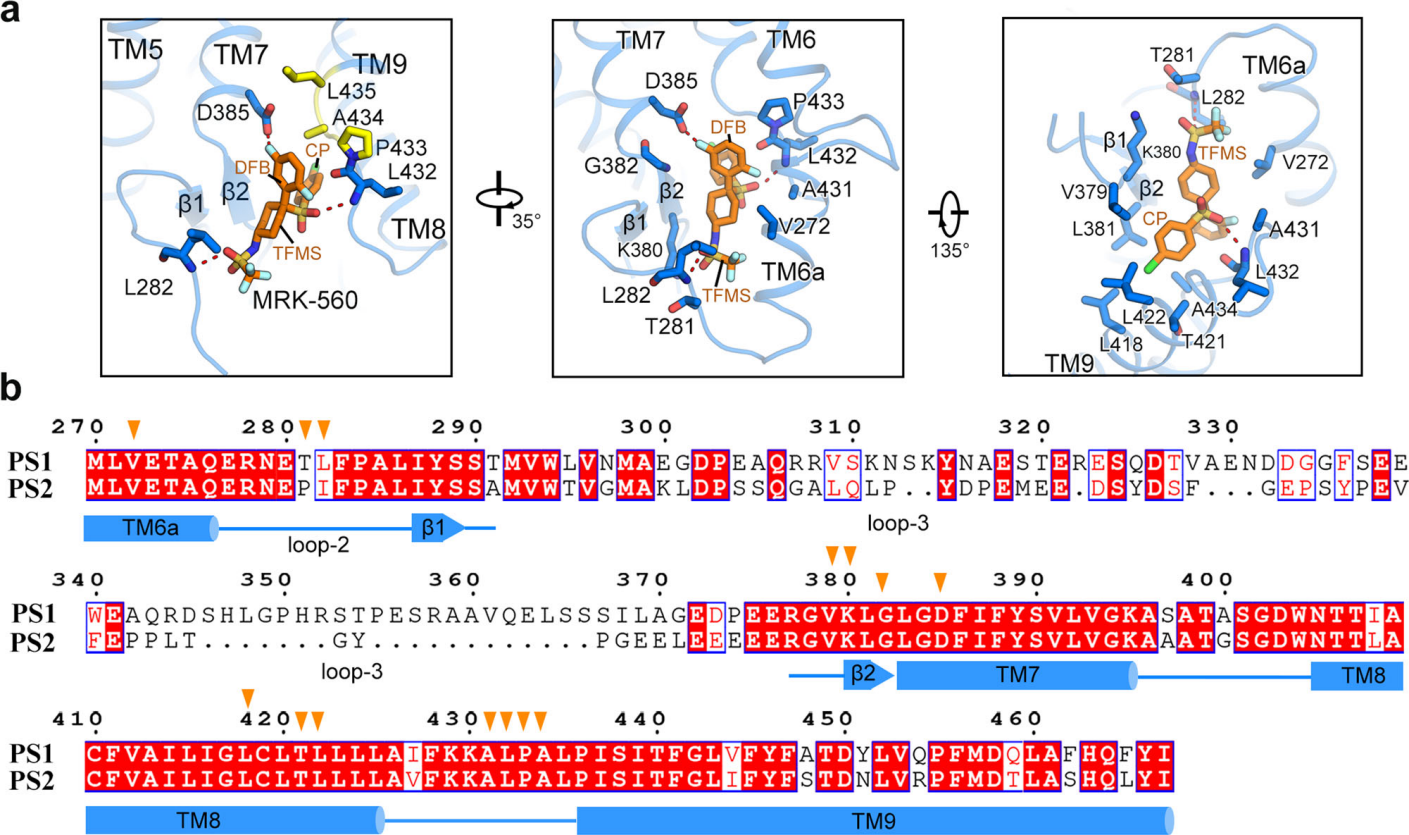
Specific recognition of MRK-560 by PS1. **a** Coordination of MRK-560 by PS1. MRK-560 forms three H-bonds with Leu432, Leu282 and Asp385 of PS1 in addition to extensive van der Waals contacts with surrounding residues. H-bonds are shown as red, dashed lines (left panel). **b** Identification of the determinant for subtype-specific recognition of MRK-560 through structure-based sequence alignment of human PS1 and PS2. Residues involved in the interactions with MRK-560 are indicated with orange triangles. Among these, Thr281 and Leu282 in PS1 are the only varied residues from those in PS2.

Next, we examined the structures to identify the molecular basis for the isoform selectivity of MRK-560 (Fig. 3a). MRK-560 forms three hydrogen bonds (H-bonds) with Asp385, Leu432 and Leu282 of PS1 (Fig. 3a, left panel). Among these, Asp385 is one of the catalytic residues of PS1, Leu432 precedes the PAL motif, and Leu282 sits on the intervening loop between TM6a and β1 of PS1 (loop-2). Ligand binding is stabilized through extensive van der Waals contacts with surrounding residues (Fig. 3a, middle panel and right panel). The difluorobenzyl (DFB) and trifluoromethanesulfonamide (TFMS) groups of MRK-560 interact with Val272, Thr281, Lys380, Gly382, Ala431, and Pro433 (Fig. 3a, middle panel), and the chlorophenyl (CP) group interact with the side chains of Val379, Leu381, Leu418, Thr421, Leu422, and Ala434 (Fig. 3a, right panel).

PS1 and PS2 share 61% sequence identity. Among the MRK-560 binding residues in PS1, only Thr281 and Leu282 are not conserved in PS2; the corresponding loci are replaced by Pro287 and Ile288 in PS2 (Fig. 3b). Both Thr281 and Leu282 are located on the loop-2 of PS1. Apart from the H-bond between the main chain amide group of Leu282 with MRK-560, the side chains of Thr281 and Leu282 directly contact the TFMS group of MRK-560 through hydrophobic interactions (Fig. 3a middle panel). To examine whether these two residues are responsible for the isoform-dependent sensitivity to MRK-560, we generated PS1 and PS2 variants with swapped residues at these two positions and examined their response to MRK-560 inhibition.

### Thr281 and Leu282 of PS1 are important for MRK-560 selectivity

**Fig. 4:**
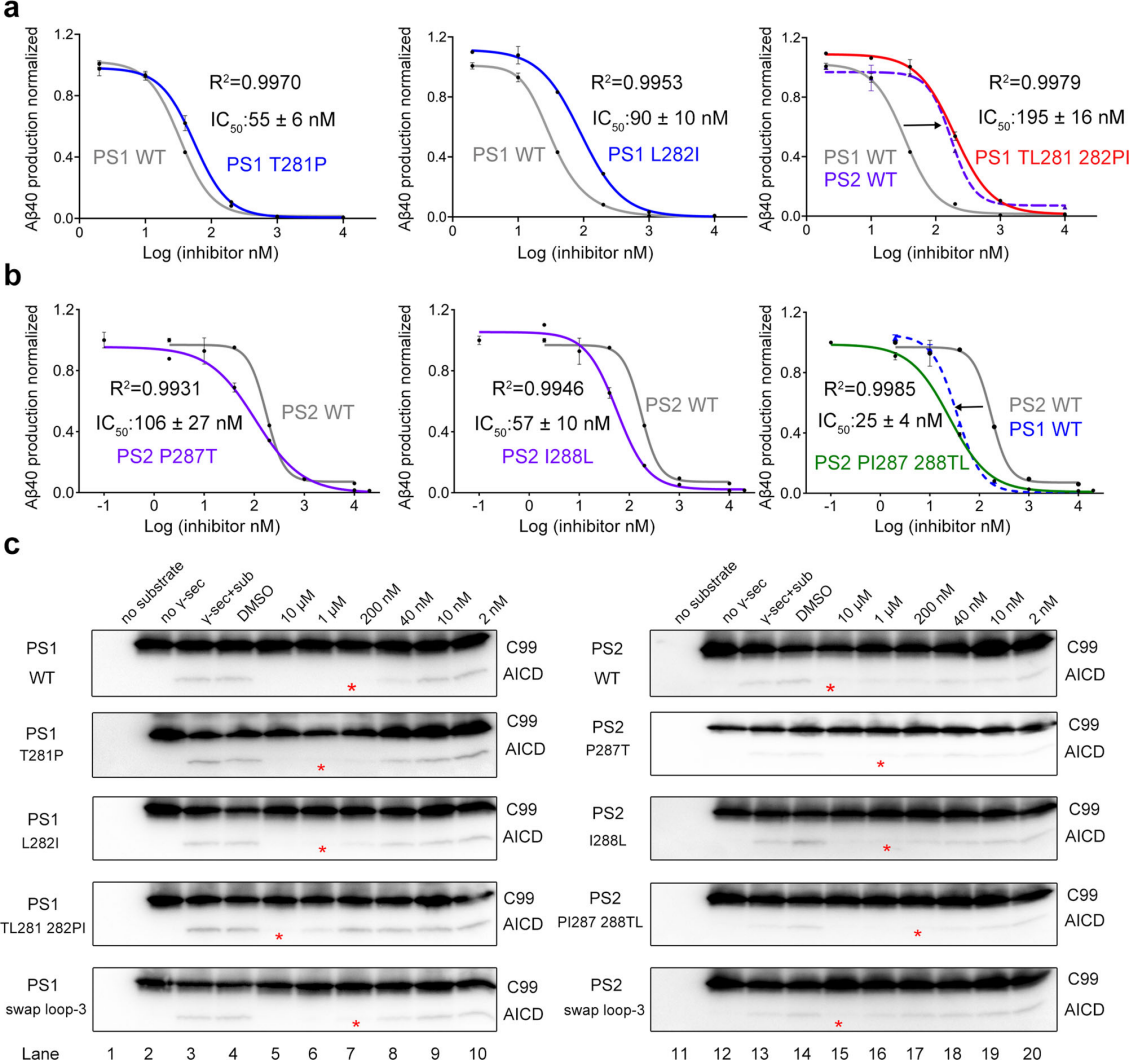
Thr281 and Leu282 of PS1 determine the isoform selectivity of MRK-560. **a–c** Individual and double residue swapping of Thr281 and Leu282 in PS1 with the corresponding Pro287 and Ile288 in PS2 alter their respective sensitives to MRK-560 accordingly. IC_50_ values for MRK-560 inhibition of the indicated PS1 (**a**) and PS2 (**b**) mutants were measured using the AlphaLISA assay that detects Aβ40 production. Please refer to Methods for experimental details. Data are presented as mean values ± SD. **c** Production of AICD by residue-swapped PS1 and PS2 mutants. The amount of AICD generated by the indicated variants in the presence of serial diluted MRK-560 is consistent with the corresponding IC_50_ shown in (**a**, **b**). In contrast, swapping of loop-3 has little influence on inhibition selectivity. The production of AICD was quantified by ImageJ. Red asterisks indicate the first concentration where the normalized production of AICD was reduced to less than 20%. Source data are provided as a Source Data file. Each cleavage assay was repeated three times. Error bars: SD.

In total we generated six variants, including single and double residue swapping of Thr281/Leu282 in PS1 with Pro287/Ile288 in PS2, respectively. MRK-560 response to the six PS1/PS2 mutant-containing complexes, each purified to homogeneity, was examined in the in vitro cleavage assay (Fig. 4).

Single point mutations T281P and L282I changed the IC_50_ of MRK-560 on PS1 from 33 ± 2 nM to 55 ± 6 nM and 90 ± 10 nM, respectively (Fig. 4a). Combination of the two mutations further lowered the variant’s sensitivity to MRK-560, with the IC_50_ of 195 ± 16 nM, which is similar to that of the WT PS2-complex (173 ± 24 nM) (Fig. 4a, right panel). Remarkably, the corresponding PS2 variants gained increased sensitivity with IC_50_ of 106 ± 27 nM, 57 ± 10 nM, and 25 ± 4 nM for P287T, I288L, and PI287-288TL, respectively (Fig. 4b). The IC_50_ of PS2 (PI287-288TL) mutant is similar to that of the WT PS1-complex (33 ± 2 nM) (Fig. 4b, right panel).

We also examined the inhibitory effect of MRK-560 on the production of AICD by the PS1/PS2 variants (Fig. 4c). For the WT PS1-complex, production of AICD was completely inhibited by 200 nM MRK-560. In contrast, AICD was still detected in the presence of 1 μM MRK-560 for the PS1 variants T281P and L282I (Fig. 4c left panel). Similar to the WT PS2 complex, PS1 TL281-282PI could not be completely inhibited for AICD production even in the presence of 10 μM MRK-560 (Fig. 4c). On the other hand, the PS2 variants showed increased sensitivity to MRK-560 (Fig. 4c right panel). Generation of AICD by PS2(PI287-288TL) or WT PS1-complex was completely inhibited with 200 nM MRK-560 (Fig. 4c).

The loop between β1 and β2 (referred to as loop-3, residues 291-375 in PS1 and 291-353 in PS2) also has lower sequence identity between PS1 and PS2 (Fig. 3b). The residues in loop-3 are invisible in our previously reported structures^14,15,26,28^. As loop-3 does not interact with MRK-560, it may have limited impact on the selective inhibition of MRK-560. Supporting this analysis, swapping loop-3 between PS1 and PS2 has no detectable effect on the sensitivity to MRK-560 (Fig. 4c). These results collectively support that the Thr281-Leu282 motif in PS1 plays a crucial role in determining the inhibitory selectivity of MRK-560.

## Discussion

Isoform-selective GSIs may afford a potential opportunity to develop therapeutics for AD and other disorders and serve as probes to delineate the function of PS1 and PS2 complexes. In this study, combining cryo-EM structural analysis and biochemical characterization, we elucidate the molecular basis for the mode of action and subtype specificity of MRK-560, a PS1-sensitive GSI. Although it remains to be investigated whether MRK-560 is effective in clinical trials as in mouse model, information derived from this study may facilitate optimization and development of subtype-selective GSIs.

Why is MRK-560, but not other GSIs, such as Avagacestat or Semagacestat, subtype specific? To address this question, we scrutinized the structures of PS1 bound to MRK-560, Avagacestat, and Semagacestat. Compared to Avagacestat or Semagacestat, one of the branches of MRK-560 is closer to loop-2 of PS1 (Supplementary Fig. 6a, b, cyan and yellow shadow). Among the three GSIs, MRK-560 is the only one that forms H-bond with residues on loop-2 (Supplementary Fig. 6a, b). As Thr281 and Leu282 in loop-2 are important for the selectivity of MRK-560, the strong association between MRK-560 and loop-2 may underlie its subtype specificity.

Structural studies of γ-secretase bound with different GSIs have identified a common GSI binding pocket comprising β1, β2, TM9, TM6a, TM6, loop-2 and PAL loop (Supplementary Fig. 6c, left panel). TM6a, loop-2 and the PAL loop are relatively mobile, manifested by their different conformations in the presence of different GSIs (Supplementary Fig. 6c, middle and right panels). The high-flexibility of this side of the pocket is crucial for accommodating ligands with various shapes (Supplementary Fig. 6c, left panel). Therefore, the elastic side of the pocket may be explored for the design of specific GSIs.

Other GSIs that are reported with PS1 selectivity include BMS299897, ELN318463, and SCH1500022 (Supplementary Fig. 7)^23,29^. All these molecules are branched with sulfone group at the center (Supplementary Fig. 7). At least one additional carbonyl/sulfone group is present in one of the branches, which may also impose different modulations of PS1 and PS2 through differential interactions with residues on loop-2 (Supplementary Fig. 7, salmon shadow). Several AD-derived mutations are mapped to loop-2, further highlighting the functional importance of this loop. Combination of the key functional groups of different GSIs may facilitate the development of lead compounds with higher specificity and selectivity.

## Methods

### Clones and plasmids

The coding DNA sequences for human PS1 or PS2 and their variants, APH-1aL, PEN-2 and NCT were individually cloned into the pMLink vector^25^. All plasmids used for transfection of mammalian cells were prepared using the EndoFree Plasmid Maxi Kit (Cwbiotech).

### Cleavage activity assays for γ-secretase

All proteins used for the activity assay were sub-cloned, expressed and purified as described^25^. Protein concentration was determined using the Bradford method. 30 nM purified wild-type (WT) γ-secretase was incubated with 12.5 µM substrate APP-C99 in a reaction buffer that contains 25 mM HEPES pH 7.4, 0.2% CHAPSO, 150 mM NaCl, 0.1% phosphatidylcholine, 0.025% phosphatidylethanolamine, and 0.00625% (w/v) cholesterol^14^. The cleavage reaction was conducted at 37 °C for 4 h. The cleaved products of these substrates were detected using a monoclonal antibody (1:1000 dilution, CW0299, CWBio) against the C-terminal Myc tag. Aβ40 production was detected using the AlphaLISA assay (PerkinElmer) as described^26^. The IC_50_ values and the inhibition curves were calculated using Prism 8.0.

### Preparation of the cryo-EM samples

WT PS1- and PS2-complex were expressed and purified from HEK293F cells as described^25^. MRK-560 at 2 mM as final concentration was incubated with 40 µM PS1 or PS2-complex at 4 °C for 0.5 h, respectively. Aliquots of 4 µl γ-secretase in complex with MRK-560 was dropped onto glow-discharged holey carbon grids (Quantifoil Au R1.2/1.3, 300 mesh). The grids were blotted for 3 s and flash-frozen in liquid ethane using Vitrobot Mark IV (FEI). The samples were imaged on an FEI 300 kV Titan Krios transmission electron microscope equipped with a Cs corrector and Gatan GIF Quantum energy filter (slit width 20 eV), recorded by a Gatan K3 Summit detector with a nominal magnification of ×81,000. A series of defocus values from −1.5 to −1.8 µm was used during data collection. Each image was dose-fractionated to 32 frames with a total electron dose of ~50 e^−^ Å^−2^ and a total exposure time of 2.56 s. AutoEMation II (developed by Lei and Frank)^30^ was used for fully automated data collection. All stacks were motion-corrected using MotionCor2 with a binning factor of 2^31^, resulting in a pixel size of 1.0825 Å. The defocus values were estimated using Gctf^32^ and dose weighing was performed concurrently^33^. The free state PS2-complex sample preparation was similar to described above.

### Cryo-EM data processing

The three datasets corresponding to apo PS2, PS1 bound MRK-560, and PS2 supplemented with MRK-560 were processed using a similar workflow (Supplementary Fig. 1). The movie stacks were manually selected after motion correction and CTF estimation respectively. 970,335 particles for apo PS2, 2,400,329 particles for PS1 bound MRK-560, and 1,939,642 particles for PS2 added MRK-560 were auto-picked using RELION-3.0^26,34,35^, respectively. After two-dimensional (2D) classification, 473,170, 458,721, and 630,931 particles were selected and subjected to three-dimensional (3D) classification respectively. In all cases, the γ-secretase EM map (EMD-3061) was low-pass filtered to 20 Å to generate an initial model^26^. The selected particles were subject to 30 iterations of global angular search 3D classification. Each of the 30 iterations has one class and a step size of 7.5°. For the last six iterations (No. 25-30) of the global search, the local angular search 3D classification was executed with a class number of four, a step size of 3.75°, and a local search range of 15°. For the last 5 iterations of the local search, particles from the good classes were merged and duplicated particles were removed. The resulting 399,077, 377,032, 515,282 particles were applied to autorefinement, yielding 3D reconstructions at 4.2-Å 3.7-Å, and 3.9-Å respectively.

Results of the initial refinement were then applied to further multi-reference 3D classification. The box size was changed from 200 to 320 pixels. 163,958, 127,676, 200,955 particles from the good classes were applied to autorefinement, resulting in 3.5-Å, 3.0-Å, and 3.0-Å reconstruction based on the Fourier shell correlation (FSC) 0.143 criterion. After postprocess, yielding 3D reconstructions at 2.9-Å, 3.4-Å and 3.0-Å respectively. The FSC curves were corrected for the effects of a soft mask using high-resolution noise substitution^36^. Local resolution variations were estimated using RELION-2.0^34^.

### Model building and structure refinement

The structures of PS1 bound MRK-560, Apo PS2 and PS2 added MRK-560 were first refined in real space using PHENIX^37^ with secondary structure and geometry restraints. 2D structures of MRK-560 was generated from the Pubchem database and initial geometry restraint was obtained through phenix.elbow new.sdf. The atomic models were manually improved using COOT. The glycans, cholesterols, and phosphatidylcholines were identified as described^26^. The final atomic model was refined in real space using PHENIX^38^, and evaluated using MolProbity^39^. The local resolution map was calculated using RELION 2.0^34^. Resolution estimations of all cryo-EM maps are based on the Fourier Shell Correlation (FSC) value of 0.143^40^. The details can be found in Supplementary Fig. 1.

### Statistical analysis of Aβ40 production

Statistical analysis of Aβ40 was performed using Prism 8 (GraphPad Software, San Diego, CA, USA). IC_50_ were determined by a four-parameter logistic regression. The statistical details can be found in Figs. 1, 4 and legends. Data are expressed as mean ± SD.

### Reporting summary

Further information on research design is available in the Nature Research Reporting Summary linked to this article.

## Supplementary information

Supplementary Information

Reporting Summary

## Data Availability

The data that support this study are available from the corresponding authors upon reasonable request. The cryo-EM maps of the structure of human, apo PS2-complex and have been deposited in the Electron Microscopy Data Bank (EMDB) with the accession code EMD-33624 (PS1-complex bound with MRK-560), EMD-33629 (apo PS2-complex), and EMD-33628 (PS2-complex with MRK-560). The cryo-EM map with the accession code EMD-3061 (human gamma-secretase complex) is used for initial model. The atomic coordinates for the corresponding model have been deposited in the Protein Data Bank (PDB) under the accession code 7Y5T (PS1-complex bound with MRK-560), 7Y5Z (apo PS2-complex), and 7Y5X (PS2-complex with MRK-560). Source data are provided with this paper.

## Source data

Source Data
