# Supplementary material for: Molecular basis for isoform-selective inhibition of presenilin-1 by MRK-560
Source: Nat Commun. 2022 Oct 22;13:6299. doi: 10.1038/s41467-022-33817-5 (PMC9587990; doi:10.1038/s41467-022-33817-5)
Supplement: Supplementary file 1 — Supplementary Information [file 41467_2022_33817_MOESM1_ESM.pdf]

# Supplementary Information

## Molecular Basis for Isoform-Selective Inhibition of Presenilin-1 by MRK-560

Xuefei Guo, Yumeng Wang, Jiayao Zhou, Chen Jin, Jiaoni Wang, Bojun Jia, Dan Jing, Chuangye Yan, Jianlin Lei, Rui Zhou, and Yigong Shi

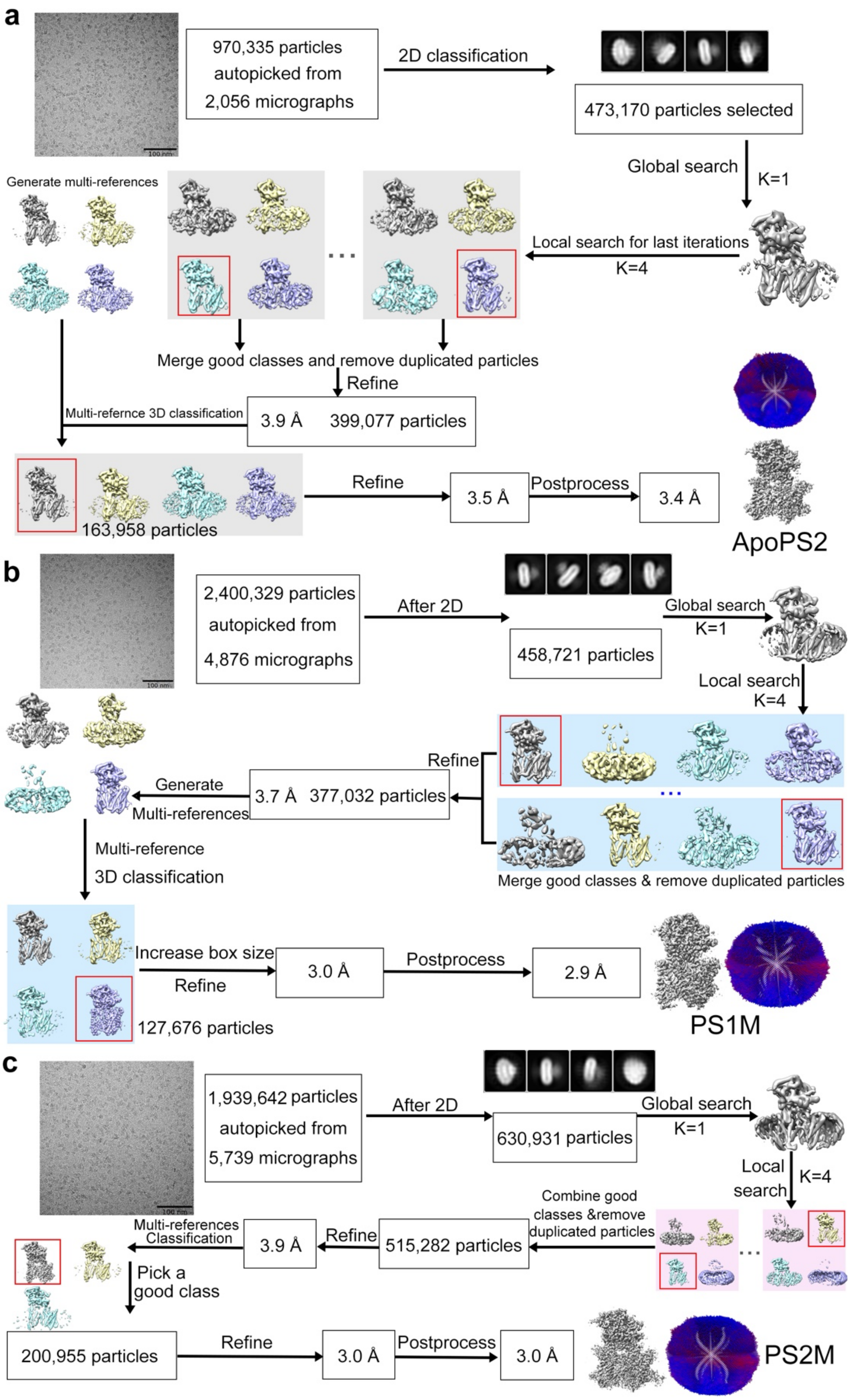


**Supplementary Fig. 1 Flowcharts of Cryo-EM data processing of ApoPS2, PS1M and PS2M respectively.** (**a-c**) Flowcharts of cryo-EM data processing of different datasets. Please refer to Methods for experimental details.

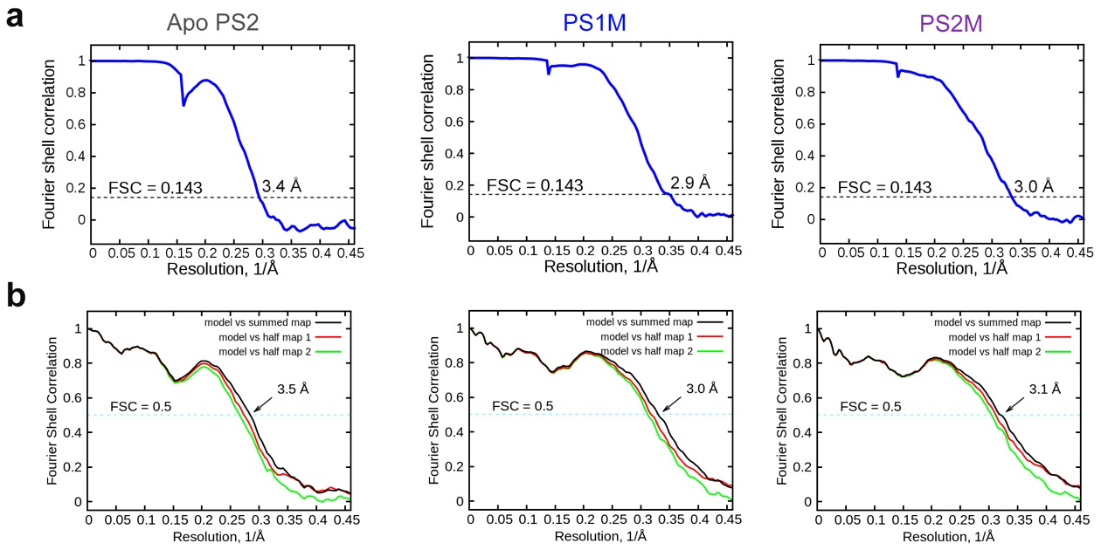


**Supplementary Fig. 2    Cryo-EM analysis of PS1- and PS2-complexes in the absence or presence of MRK-560, respectively.** (**a**) The resolutions of the three indicated 3D EM reconstructions corresponding to the 0.143 FSC curve. (**b**) The FSC curves of the refined model versus the maps that it is refined against (black); of the model refined in the first of the two independent maps used for the FSC calculation versus that same map (red); and of the model refined in the first of the two independent maps versus the second independent map (green). The small difference between the red and green curves indicates that the refinement did not suffer from overfitting.

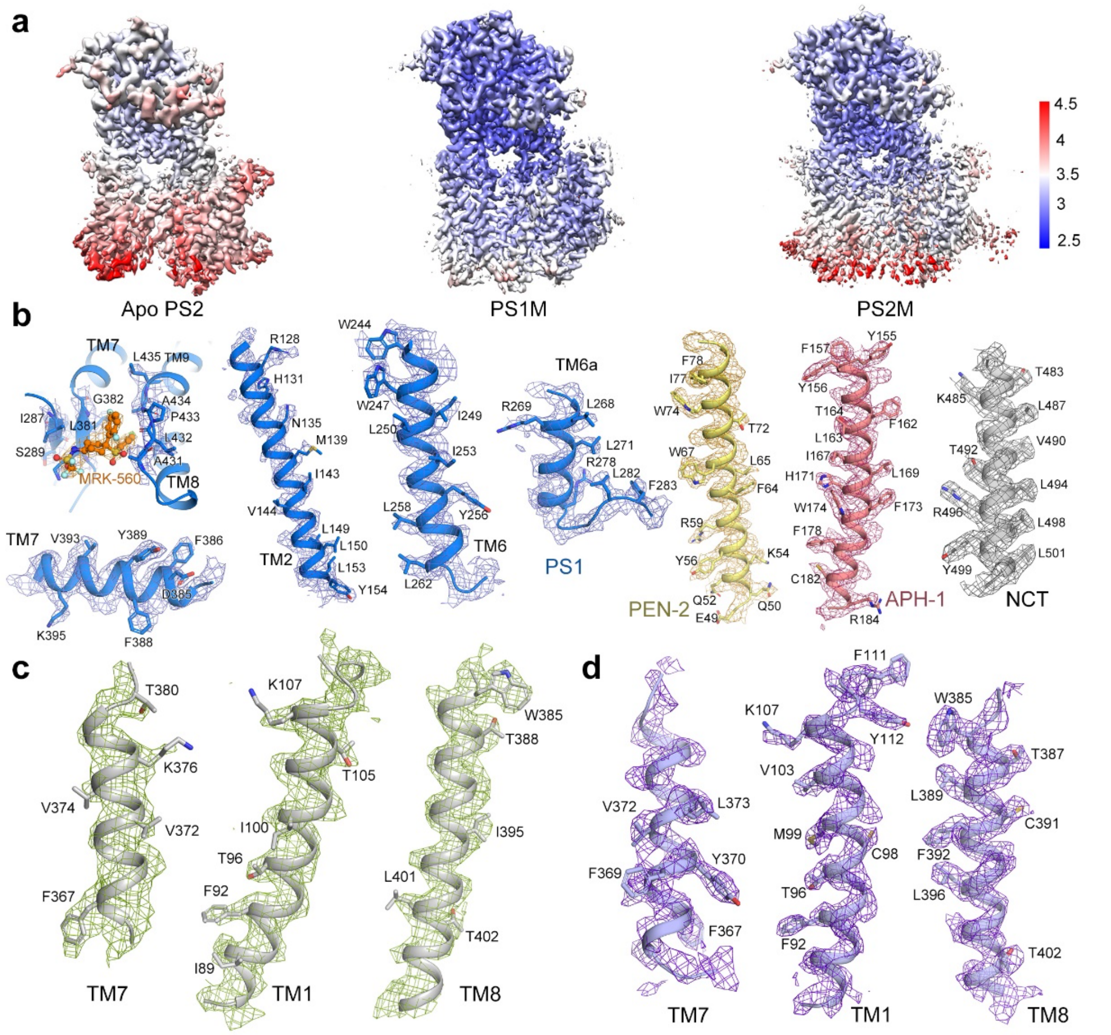


**Supplementary Fig. 3 Representative densities for the PS1- and PS2-complexes.** (**a**) Local resolution distribution of the final reconstruction for the PS1- and PS2-complexes, estimated by RELION-2.0. (**b**) EM densities for representative segments in the PS1-complex bound to MRK-560. (**c**) Representative densities of the ligand-free PS2-complex. (**d**) Representative densities of the PS2-complex treated with MRK-560. All densities shown here are contoured at 5σ.

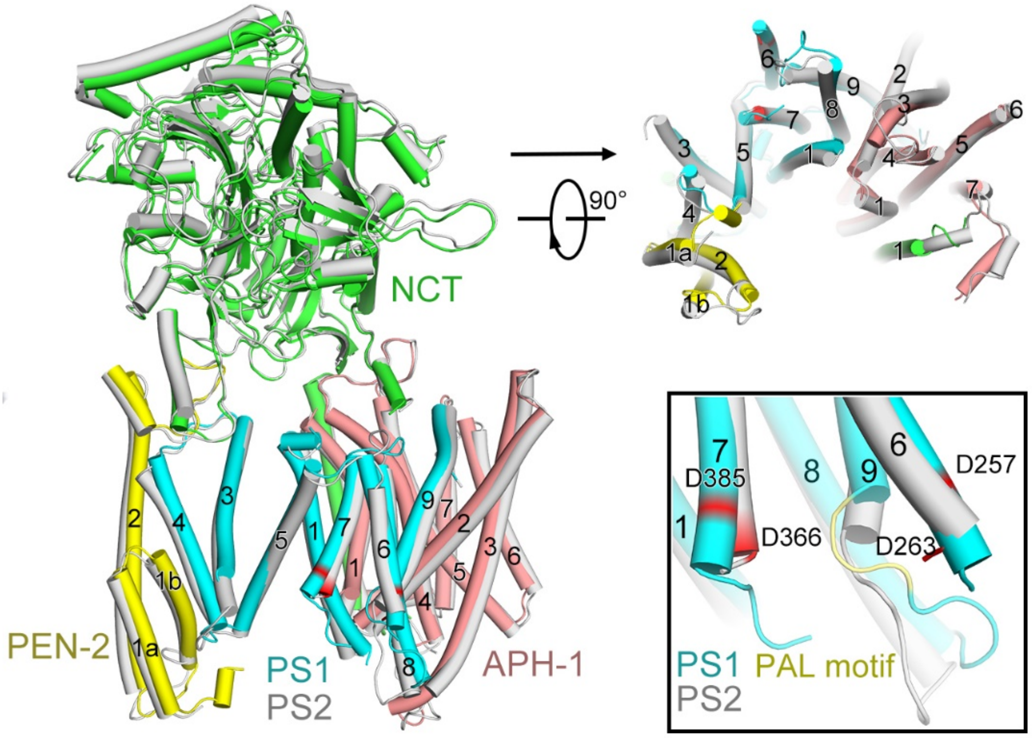


**Supplementary Fig. 4  Structural comparison of the ligand-free PS1- and PS2-complexes.** *Left*: Comparison of overall models between PS1-complex and PS2-complex in free states. The overall conformation of PS1-complex is almost identical to that of PS2. *Right*: Subtle variations occur to TM6 and TM7, where the catalytic residues and the PAL-loop reside. *Inset*: The positions of the catalytic Asp residues, D257/D385 in PS1 and D263/D366 in PS2, are colored red. The PAL motif in PS1 is colored yellow.

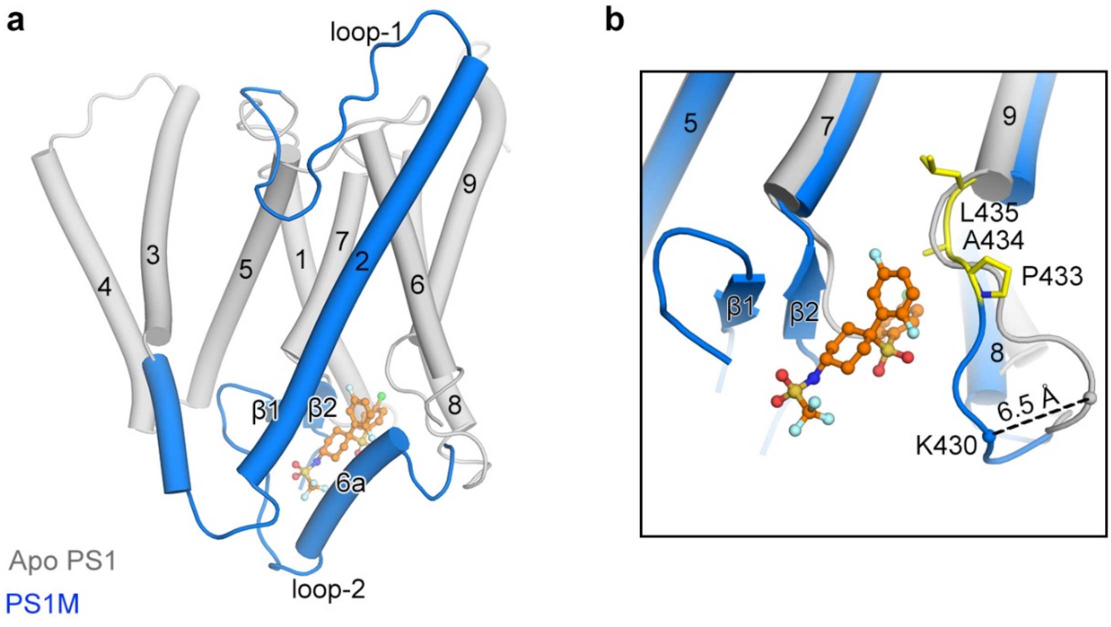


**Supplementary Fig. 5 Conformational changes of PS1 upon MRK-560 binding.** (**a**) Profound structural rearrangement of PS1 upon MRK-560 binding. The structural elements that are observed only in the presence of MRK-560 is colored blue. (**b**) Binding of MRK-560 induces displacement of the PAL loop (yellow) and formation of an anti-parallel β-hairpin.

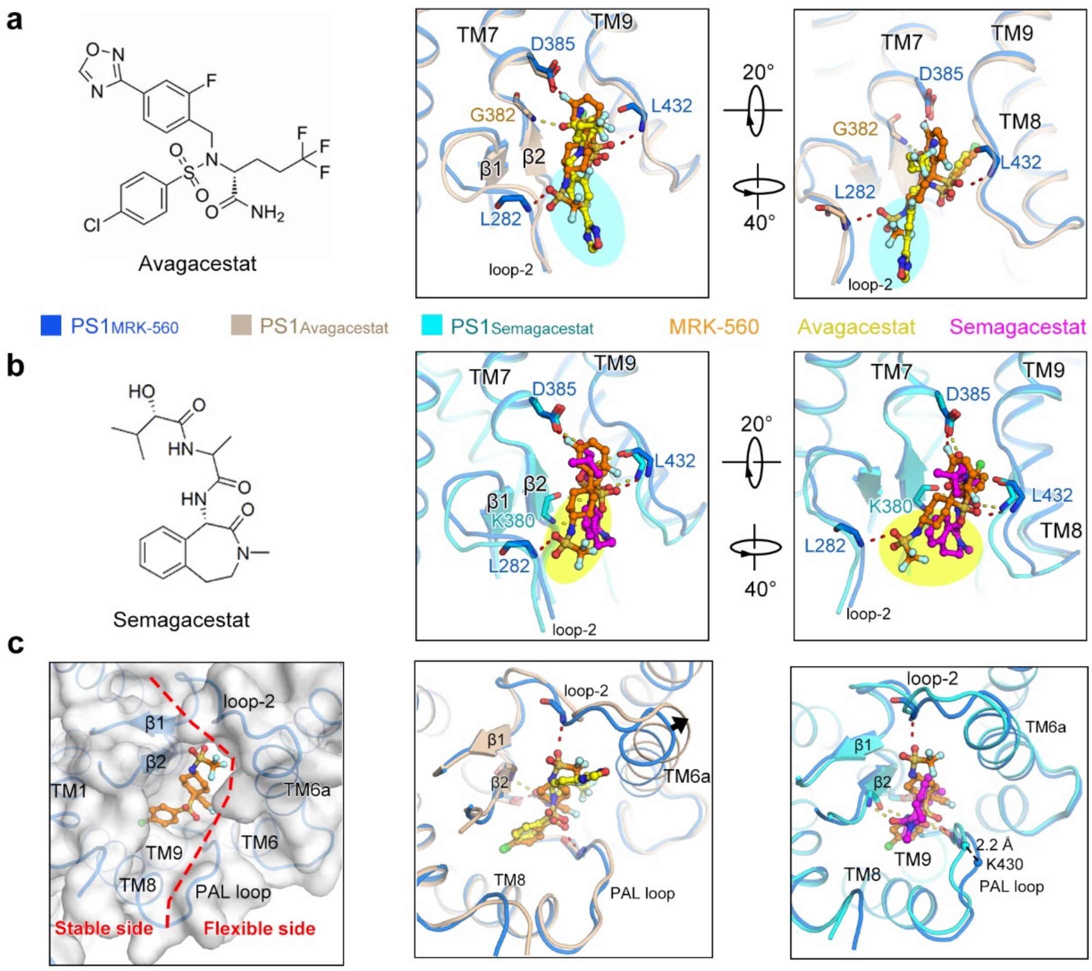


**Supplementary Fig. 6 Structural comparison of the PS1 complex bound to MRK-560 and two other non-transition state analog (non-TSA) GSIs.** (**a**) A close-up comparison of the GSI-binding cavities between MRK-560-bound and Avagacestat-bound PS1. The chemical structure of Avagacestat is shown on the left. MRK-560 is H-bonded with Asp385, Leu282 and Leu432 of PS1, whereas Avagacestat forms only one H-bond with Gly382 of PS1 (middle panel). Branches of MRK-560 and Avagacestat (shadowed pale cyan) are at different distances with loop-2, with MRK-560 closer to loop-2 (right panel). (**b**) Structural comparison of the MRK-560-bound and Semagacestat-bound PS1. The chemical structure of Semagacestat is shown on the left. Five H-bonds are found between Semagacestat and PS1 residues, including Asp385, Leu432 and Lys380 (middle panel). One branch of MRK-560

(shadowed yellow) is closer to loop-2 compared to Semagacestat (right panel). (**c**) One side of the GSI binding pocket is relatively mobile (left panel). Compared to MRK-560-bound PS1, TM6a is pushed further away by Avagacestat from the inhibitor binding pocket, resulting in a more relaxed conformation (middle panel). The PAL loop of PS1 exhibits slightly different conformations in the presence of MRK-560 and Semagacestat, indicated by the 2.2 Å-displacement of the Cα atom of Lys430 in the superimposed structures (right panel).

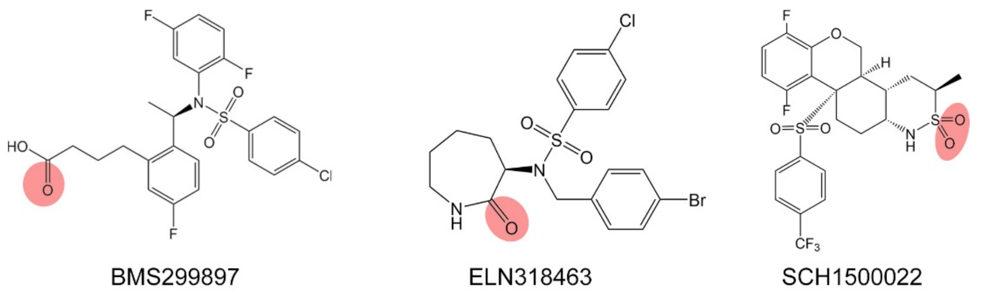


**Supplementary Fig. 7 PS1-selective GSIs share similar branched features to MRK-560.**

Chemical structures of three other representative PS1-selective GSIs are shown. All molecules are branched. At least one of their branches contains a carbonyl/sulfone group (shadowed salmon), which is likely to interact with residues on loop-2.
